# The role of exosomes in bladder cancer immunotherapy

**DOI:** 10.1016/j.jncc.2025.04.001

**Published:** 2025-05-02

**Authors:** Mohammad Mousaei Ghasroldasht, Piyush K. Agarwal

**Affiliations:** Department of Surgery, Section of Urology, University of Chicago, Chicago, IL, United States

**Keywords:** Bladder cancer, Cancer immunotherapy, Exosome, Exosome based immunotherapy

## Abstract

Bladder cancer remains a significant global health challenge, requiring repeated treatments and surveillance and potentially morbid therapies, particularly in advanced and recurrent stages. Exosomes, small extracellular vesicles central to intercellular communication, have emerged as innovative tools in cancer diagnostics, prognosis, and therapy. Their role in modulating the immune response and the tumor microenvironment makes them particularly attractive for cancer immunotherapy. This review provides a comprehensive overview of exosome biology, with a focus on their role in immune modulation and potential therapeutic applications. We explore the progress and challenges of exosome-based immunotherapy in cancer, followed by a discussion on the current state of bladder cancer immunotherapy. Additionally, we highlight the roles of exosomes in bladder cancer, emphasizing their diagnostic and prognostic applications. Despite promising preclinical studies and a growing number of clinical trials in other cancers, exosome-based therapies remain underexplored in bladder cancer. We discuss the current clinical trials related to exosomes in bladder cancer and propose their potential future role in immunotherapy. Finally, we address the challenges and opportunities in translating exosome-based therapies from bench to bedside, emphasizing the need for further preclinical and clinical investigations. This review emphasized the potential of exosome-based immunotherapy as a transformative approach for bladder cancer diagnosis and treatment.

## 1 Introduction

The concept of immunotherapy dates back to the late 19th century when Dr. William Coley first used bacterial toxins in 1891 to stimulate the immune system against cancer, laying the foundation for modern immunotherapy.^1^ In the 20th century, breakthroughs such as the discovery of interferons (1957) and interleukins (1976) expanded the understanding of immune regulation.^2^^,^^3^ The approval of the first monoclonal antibody therapy (rituximab) in 1997 marked a significant milestone in targeted immunotherapy.^4^ The early 2000s saw the emergence of immune checkpoint inhibitors, with the FDA approval of anti-CTLA-4 (cytotoxic T lymphocyte-associated protein 4) (ipilimumab) in 2011 and anti-PD-1/PD-L1 (programmed cell death protein 1) (nivolumab, pembrolizumab) in 2014, revolutionizing cancer treatment by enhancing the body’s natural immune response.^5^ Around the same time, adoptive cell transfer (ACT) therapies, including chimeric antigen receptor (CAR) T-cell therapy, gained traction, leading to the FDA approval of the first CAR-T therapy (tisagenlecleucel) in 2017.^6^ Despite significant advances, challenges such as immune evasion, therapy resistance, and adverse events still limit the effectiveness of current immunotherapies, particularly in solid tumors.^7^ Tumors employ various immune evasion strategies, such as upregulating immune checkpoint proteins (e.g., PD-L1, CTLA-4), recruiting regulatory T cells (Tregs) and myeloid-derived suppressor cells (MDSCs), and secreting immunosuppressive cytokines that inhibit effective immune responses. These mechanisms not only allow tumor cells to evade immune surveillance but also contribute to resistance against conventional therapies, including chemotherapy, radiation, and immunotherapy. As a result, overcoming immune suppression within the tumor microenvironment (TME) is critical for improving cancer treatment outcomes.

Over the past two decades, numerous modifications have been made to immunotherapy strategies to address these challenge and boost anti-tumor immunity. These advancements include the development of immune checkpoint inhibitors (ICIs), ACT, oncolytic virus therapy (OV), and cancer vaccines. ICIs block specific immune checkpoints, which are normally used to prevent immune responses from damaging healthy tissues.^8^ However, tumors exploit these pathways by expressing checkpoint proteins like CTLA-4, PD-1, and its ligand (PD-L1), which help cancer cells evade immune detection and promote T-cell exhaustion.^9^, ^10^, ^11^ ICIs targeting checkpoint proteins have shown significant success in cancers such as melanoma, non-small-cell lung cancer (NSCLC), and bladder cancer (BC).^12^, ^13^, ^14^ Despite these successes, response rates remain variable, and not all patients benefit from these therapies. Additionally, immune-related adverse events (irAEs), such as myocarditis and atherosclerosis, complicate ICI use, emphasizing the need for careful patient monitoring.^15^^,^^16^ ACT involves isolating, expanding, and, in some cases, genetically modifying a patient’s immune cells (e.g., T cells or natural killer (NK) cells) before reintroducing them into the body to fight cancer.^17^ ACT has shown success in hematologic cancers like leukemia and lymphoma, but its application to solid tumors is challenged by the immunosuppressive TME.^18^, ^19^, ^20^ Solid tumors can induce immune resistance through mechanisms such as immune checkpoint activation, T-cell exhaustion, and the presence of Tregs.^21^^,^^22^ To overcome these challenges, researchers are exploring strategies such as combining ACT with ICIs or enhancing the persistence of infused cells with cytokines like interleukin-2 (IL-2).^23^, ^24^, ^25^ OVs represent an innovative class of therapies that selectively infect and destroy tumor cells while sparing healthy tissues.^26^ These viruses, which can be naturally occurring or genetically engineered, enhance tumor specificity and immune-stimulating properties. OVs work by lysing tumor cells and releasing tumor-associated antigens, stimulating both innate and adaptive immune responses. This process can convert “cold” tumors, which are poorly immunogenic, into “hot” tumors, more susceptible to immune attack.^27^ An example is Talimogene laherparepvec (T-VEC), a genetically modified herpes simplex virus (HSV-1) approved for melanoma treatment.^28^ T-VEC expresses granulocyte-macrophage colony-stimulating factor (GM-CSF), which recruits dendritic cells (DCs) and other antigen-presenting cells (APCs) to the site of infection, further enhancing the immune response.^28^ Other viruses, such as adenoviruses, measles virus, and reoviruses, are also being explored for their oncolytic potential.^7^^,^^29^, ^30^, ^31^ Despite early successes, challenges remain, especially concerning optimal delivery methods and resistance mechanisms in solid tumors.^32^ Cancer vaccines aim to activate the immune system by presenting tumor-specific antigens (TSAs) to APCs, which then stimulate T cells to target and destroy cancer cells.^33^ Although cancer vaccines have the potential to induce strong, targeted immune responses, they face challenges such as limited efficacy in large tumors, patient variability, and the complexity of the TME.^34^ Combining cancer vaccines with other immunotherapies, like ICIs, may enhance their effectiveness and address some of these challenges.^34^^,^^35^

Despite these advancements, one of the major obstacles in current immunotherapy methods, especially for solid tumors, is the immunosuppressive TME. The TME contains various factors that impair immune responses, including suppressive immune cells, soluble inhibitory factors, and metabolic barriers that prevent effective tumor eradication.^36^, ^37^, ^38^ Therefore, researcher are exploring new strategies as novel tools to overcome these challenges and enhance therapeutic efficacy. A growing body of research suggests exosomes, small extracellular vesicles (EVs) secreted by both tumor and immune cells, play a pivotal role in shaping the TME and influencing cancer progression by transferring bioactive molecules such as antigens, cytokines, and immune checkpoint proteins, which can modulate immune cell function. Exosomes, ranging from 30 to 160 nanometers in diameter, carry molecular components such as proteins, lipids, nucleic acids, and glycoconjugates, reflecting the characteristics of their originating cells.^39^ These vesicles facilitate intercellular communication, impacting various aspects of cancer immunity. Tumor-derived exosomes (TDEs) can promote immune evasion by transferring immunosuppressive molecules, while immune cell-derived exosomes can enhance anti-tumor immunity by delivering tumor-associated antigens or immunostimulatory molecules.^40^, ^41^, ^42^

Unlike traditional immunotherapies that rely on broad immune activation, exosomes provide a targeted, less toxic approach by delivering immune-modulating cargo directly to tumor cells, thereby enhancing therapeutic efficacy while minimizing systemic side effects. Additionally, exosomes can be engineered to carry multiple therapeutic agents, making them highly recommended for addressing the complex challenges of cancer treatment. Exosomes can modulate the immune response by carrying and delivering bioactive molecules, including tumor antigens, ICIs, and cytokines, directly into the TME. By engineering exosomes to carry PD-1/PD-L1 inhibitors or immune-stimulating molecules, these vesicles can enhance checkpoint blockade therapy and potentially improve patient responses to ICIs.^43^ Additionally, exosomes derived from immune cells, such as DCs or NK cells, can be harnessed to boost anti-tumor immunity and augment ACT therapies. These properties position exosome-based therapies as promising adjuncts to conventional immunotherapy, offering potential solutions to overcome immune evasion and resistance mechanisms in cancer treatment. However, despite these advantages, exosome-based therapies are still considered a relatively new area of research, with ongoing clinical trials to assess their efficacy and safety.

In this review, we will explore the multifaceted role of exosomes in immune modulation, focusing on their impact on cancer immunity. We will begin by discussing exosome biogenesis and composition, followed by their dual roles in immune suppression and activation within the TME. The review will then highlight the emerging potential of exosome-based immunotherapies, particularly in bladder cancer (BC). We will examine how exosomes can be utilized to overcome immune suppression in BC and enhance the efficacy of existing immunotherapies. Additionally, we will explore the role of exosomes in BC pathogenesis, their potential as non-invasive diagnostic tools, and their therapeutic applications. Finally, we will consider the future of exosome-based therapies in BC, addressing current challenges and prospects for expanding these strategies to treat other solid tumors.

## 2 Exosome biology and immune modulation

Exosomes are small EVs, typically 30–160 nm in diameter, that are released by nearly all cell types. They originate from the endosomal system through the formation of multivesicular bodies (MVBs) and play a pivotal role in intercellular communication by transferring bioactive molecules such as proteins, lipids, and nucleic acids between cells. Exosomes are involved in various physiological and pathological processes, including immune regulation, tissue repair, and disease progression.^44^ In the context of cancer, exosomes contribute to both immune suppression and immune activation by modulating key components of the immune system.^45^ They can facilitate immune evasion by promoting Tregs, inhibiting cytotoxic T lymphocytes (CTLs), and suppressing DCs function. Conversely, they also have the potential to enhance immune responses by presenting tumor antigens and stimulating immune activation. Understanding the dual role of exosomes in immune modulation is critical for harnessing their therapeutic potential in cancer treatment.

### 2.1 Exosome biogenesis and composition

Exosomes play a key role in intercellular communication by carrying proteins, lipids, and nucleic acids, and are involved in various physiological processes such as immune modulation, signal transduction, and antigen presentation.^40^ Exosome biogenesis is a highly regulated process involving several steps: cargo sorting into MVBs, transport to the plasma membrane, and fusion with the membrane to release the vesicles.^46^ This process is influenced by factors such as cell type, the surrounding microenvironment, and the specific cargo to be sorted, which contributes to the heterogeneity of exosomes. Notably, cancer cells can hijack these mechanisms, altering the composition and function of exosomes to promote tumor progression and metastasis.

Exosomes contain a diverse array of biomolecules, which reflect the cellular origin of the vesicles and their physiological state. The main components of exosomes are crucial for their biological functions and intercellular communication:

**Proteins:** The protein content of exosomes is highly variable, depending on the cell of origin and its functional state. Exosomal proteins can be broadly categorized into housekeeping proteins, membrane-associated proteins, and cargo proteins.^47^, ^48^, ^49^ Key proteins found in exosomes include heat shock proteins (e.g., Hsp70 and Hsp90), tetraspanins (e.g., CD9, CD63, and CD81), adhesion molecules (e.g., integrins and selectins), and enzymes (e.g., proteases and kinases).^48^^,^^49^ These proteins are involved in vesicle biogenesis, membrane fusion, and the sorting of cargo. Specific proteins such as Alix and TSG101 are essential for the formation of MVBs and for selecting cargo proteins to be incorporated into exosomes.^50^ Additionally, exosomes often carry surface markers that reflect the cell type from which they originate, enabling the tracking and identification of exosomes in various biological contexts.^51^

**Lipids:** Exosomal lipids play a crucial role in membrane structure, stability, and the functional properties of exosomes. Common lipid classes found in exosomes include sphingomyelin, phosphatidylcholine, and cholesterol, which are important for maintaining membrane integrity.^52^^,^^53^ Lipids such as ceramide and lysobisphosphatidic acid (LBPA) are enriched in the exosomal membrane and are involved in the biogenesis of vesicles, particularly the formation of intraluminal vesicles (ILVs) within MVBs.^54^ The lipid composition of exosomes can vary depending on the cell type and its functional state, and exosomes from cancer cells may have distinct lipid profiles that influence their interactions with target cells and their ability to mediate cellular signaling.

**Nucleic acids:** Exosomes are also known to carry various types of nucleic acids, including mRNA, miRNA, and non-coding RNAs and in certain contexts, small DNA fragments.^55^ RNA molecules serve as important messengers in intercellular communication, influencing gene expression in recipient cells and modulating processes such as proliferation, apoptosis, and immune responses.^56^^,^^57^ DNA fragments, particularly in the context of cancer, can carry oncogenes or tumor suppressor genes to distant cells, contributing to metastasis and tumor progression.^58^^,^^59^

**Other molecules:** In addition to proteins, lipids, and nucleic acids, exosomes may contain other molecules such as metabolites, cytokines, and transcription factors that further contribute to their biological activity.^60^^,^^61^

The composition of exosomes is dynamic and can be influenced by various factors, including cellular differentiation, activation, or stress.^39^^,^^58^^,^^62^^,^^63^ This makes exosomes highly context-dependent, with their cargo providing valuable insights into the physiological state of the donor cell. Importantly, the cargo within exosomes can change in disease states, such as cancer, cardiovascular diseases, and neurodegenerative disorders, making exosomes potential biomarkers for diagnosis and therapeutic targets.^64^, ^65^, ^66^, ^67^, ^68^

### 2.2 Exosome-induced immune suppression in cancer

**Fig. 1:**
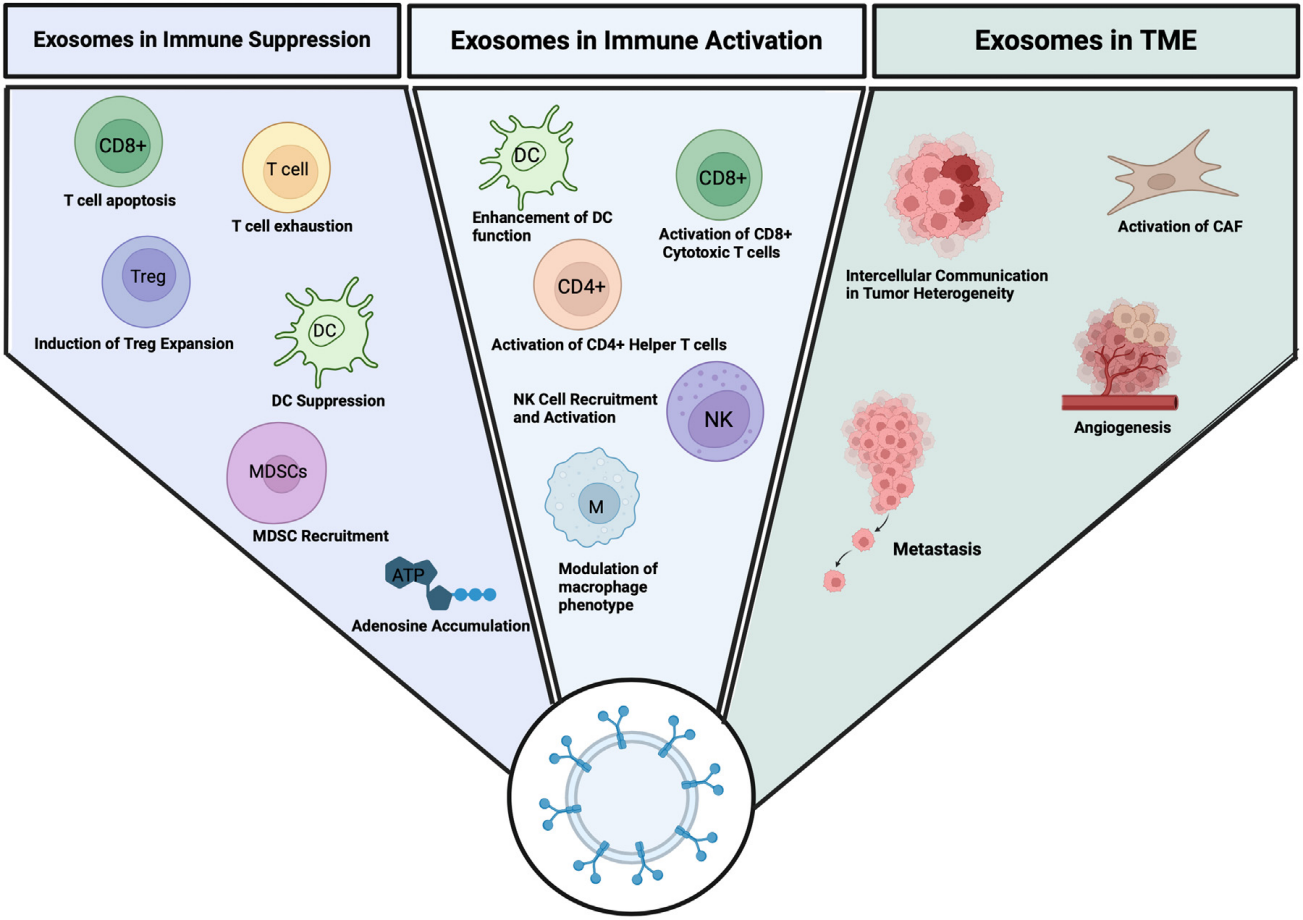
Roles of exosomes in immune modulation and the TME. Exosome-induced immune suppression: Immune suppression by inducing apoptosis in CD8^+^T cells, suppressing T cell activation (e.g., via CD69 inhibition), promoting Treg expansion through TGF-β signaling, impairing DC maturation, recruiting MDSCs, and increasing adenosine accumulation, which dampens T cell responses. Exosome-induced immune activation: Activation of immune responses by maturing DCs, presenting TSAs via MHC molecules to stimulate CD4^+^ and CD8^+^ T cells, recruiting and activating NK cells, polarizing macrophages toward an M1 phenotype, and enhancing T cell proliferation through immunostimulatory cytokines. Role of exosomes in the TME: Exosomes contribute to TME remodeling by activating CAFs with growth factors like TGF-β, facilitating intercellular communication to drive tumor heterogeneity, promoting angiogenesis through delivery of VEGF and miRNAs, and supporting metastasis via EMT and premetastatic niche preparation. CAF, cancer-associated fibroblasts; EMT, epithelial-to-mesenchymal transition; MDSCs, myeloid-derived suppressor cells; MHC, major histocompatibility complex; TME, tumor microenvironment; TSA, tumor-specific antigen.

In the TME, exosomes have been shown to play a central role in immune suppression, helping tumors evade immune surveillance in a variety of ways (Fig. 1). TDEs can deliver TSAs to APCs, triggering both innate and adaptive immune responses. The ability of TDEs to present antigens via major histocompatibility complex (MHC) molecules and stimulate both cytotoxic T cells (CD8^+^) and helper T cells (CD4^+^) offers a promising approach to activate the immune system to target and eliminate tumors.^69^ However, TDEs also carry immune-modulatory molecules that can suppress immune responses and promote immune evasion, contributing to tumor progression and resistance to immunotherapy.

#### 2.2.1 T cell apoptosis and inhibition of T cell activation

When CD8^+^ T cells come into contact with TDEs, they can undergo apoptosis, resulting in a reducing in the number of functional T cells available to fight the tumor.^70^^,^^71^ A kay marker of T cell activation is CD69, which is upregulated upon T cell activation.^72^ TDEs have been shown to suppress CD69 expression on T cells, thereby preventing their full activation and reducing T cell responses, which contributes to the failure of the immune system to effectively target and eliminate tumor cells.^73^ Additionally, exosomes from various cancers, including melanoma, colorectal cancer, and pancreatic cancer, have been shown to carry pro-apoptotic molecules, such as FasL, which trigger apoptosis in activated T cells.^42^^,^^74^ TDEs from nasopharyngeal carcinoma (NPC) have also been found to contain galectin-9, a ligand for Tim-3, which induces apoptosis in mature Th1 and CD4^+^ T cells, further dampening T cell function.^75^ This process not only limits the immune response but also facilitates resistance to immunotherapy, particularly in cases where ICIs attempt to reactivate T cells. By eliminating activated T cells or inhibiting their activation, exosomes effectively prevent the therapeutic effects of T cell-targeted treatments.

#### 2.2.2 Promotion of TREG expansion

TDEs not only impair effector T cell responses but also promote the differentiation of CD4^+^ T cells into Tregs, a population of immunosuppressive cells that inhibit immune responses, including the activity of CD8^+^ cytotoxic T cells.^76^^,^^77^ This shift toward a higher population of Tregs in the TME is largely driven by signaling molecules, including TGF-β, IL-10 and miR-155, which are packaged into exosomes and delivered to CD4^+^ T cells.^78^, ^79^, ^80^ The expansion of Treg further contributes to immune suppression by inhibiting the function of other immune cells, promoting tumor cell survival, and dampening anti-tumor immunity.^81^^,^^82^ The increased Treg population is a major barrier to therapeutic strategies, including ICIs, which rely on the activation of effector T cells to target and destroy tumor cells. Tregs in the TME actively limit the effectiveness of such treatments, making them less successful.^83^ Thus, Tregs play a central role in limiting the activation of effector immune cells, ensuring that the immune response does not adequately target tumor cells.

#### 2.2.3 DC suppression and impaired antigen presentation

Exosomes can suppress DCs, impairing their ability to present tumor antigens to T cells and hindering immune responses.^84^ TDEs have been shown to inhibit the differentiation of myeloid precursor cells into functional CD11c^+^ DCs and even induce apoptosis in mature DCs.^85^ This disruption hampers the ability of DCs to migrate to lymph nodes, where they typically interact with T cells and initiate immune responses. As a result, the immune system’s ability to recognize and target TSAs is compromised, contributing to immune tolerance and the failure to recognize the tumor as a threat. This impairment of DC function contributes significantly to resistance against immunotherapy, especially in therapies that rely on antigen presentation and activation of T cell responses, such as tumor vaccines and adoptive T cell therapy.

#### 2.2.4 Adenosine accumulation

Exosomes from tumor cells are enriched in enzymes such as CD73, which convert extracellular ATP into adenosine.^86^ Adenosine is a potent immunosuppressive molecule that acts through specific receptors on T cells, suppressing their activation and proliferation.^87^^,^^88^ Notably, adenosine can induce anergy in CD8^+^ T cells, a state of unresponsiveness, and inhibit the production of essential cytokines such as IL-2, which are necessary for T cell expansion and function.^89^^,^^90^ Additionally, adenosine promotes the differentiation and expansion of Tregs, further exacerbating immune suppression.^91^ By enhancing the Treg population and inhibiting T cell activation, adenosine contributes significantly to immune evasion in the TME. This accumulation of adenosine in the TME is a known mechanism of resistance to immunotherapies, especially ICIs like PD-1 and CTLA-4 inhibitors, which aim to reactivate exhausted T cells. Adenosine accumulation limits the effectiveness of these therapies by inducing T cell anergy and Treg expansion.^86^

#### 2.2.5 MDSC recruitment

TDEs also contribute to immune suppression by promoting the recruitment of MDSCs, a heterogeneous population of immature myeloid cells that play a critical role in tumor-induced immune suppression.^92^ Exosomes released by tumor cells have been shown to induce the differentiation of bone marrow-derived myeloid cells into MDSCs.^93^ These exosomes carry molecules such as prostaglandin E2 (PGE2) and TGF-β, which promote the accumulation of MDSCs in the TME.^93^ Once in the tumor, MDSCs produce immunosuppressive cytokines like IL-6, VEGF, and arginase-1, which dampen T cell responses and promote tumor growth.^93^, ^94^, ^95^ The presence of MDSCs in the TME is associated with poor clinical outcomes, as they not only suppress anti-tumor immunity but also foster an environment that supports tumor progression, metastasis, and resistance to therapies.^96^ MDSCs play a key role in the resistance to both chemotherapy and immunotherapy by directly suppressing T cell responses and promoting an immunosuppressive microenvironment that hinders the effectiveness of therapeutic interventions.^97^

#### 2.2.6 T cell exhaustion

Exosomes released by tumor cells can also play a significant role in driving T cell exhaustion, a state where T cells lose their effectiveness in responding to cancer cells.^98^ This phenomenon is marked by a reduced ability to produce cytokines and an upregulation of inhibitory receptors such as PD-1, TIM-3, and CTLA-4.^99^ Exosomes have been shown to contribute to this dysfunction by transferring specific molecules, including miR-21–5p, miR-155, and miR-34a, that promote immune suppression.^100^, ^101^, ^102^ For example, exosomes can deliver miRNAs that target immune cells, leading to the activation of anti-inflammatory macrophages and the suppression of T cell activity. Additionally, exosomes can carry immune checkpoint molecules like PD-L1 and galectin-9, which bind to their corresponding receptors on T cells (e.g., PD-1 and Tim-3), further promoting T cell exhaustion.^103^ This interaction prevents the T cells from performing their role in combating tumors, resulting in a progressively weakened immune response. Exhausted T cells are less responsive to immunotherapies that aim to reactivate T cells, such as PD-1/PD-L1 blockade therapies. Exosomes play a critical role in accelerating T cell exhaustion and thereby contribute to therapy resistance.

#### 2.2.7 Metabolic reprogramming of T cells

Immune cells, particularly effector T cells, rely on a metabolic shift to support their activation and proliferation in response to tumor-induced stress, such as low oxygen and nutrient availability.^104^ However, TDEs can disrupt this metabolic reprogramming, undermining the ability of T cells to mount an effective immune response. By influencing key metabolic pathways, such as glycolysis, exosomes limit the availability of glucose and other nutrients essential for T cell activation.^105^ This metabolic reprogramming not only impairs T cell function but also exacerbates the immune suppressive environment, allowing the tumor to persist and grow despite the presence of immune cells. This disruption of metabolic reprogramming is another critical factor contributing to resistance to immunotherapy, as effector T cells rely on efficient metabolic pathways to function effectively in response to cancer treatment.

### 2.3 Exosome-induced immune activation in cancer

While exosomes are often associated with immune suppression, they also have the capacity to activate immune responses, particularly in the context of antigen presentation. TDEs deliver TSAs to APCs, activating both CD4^+^ and CD8^+^ T cells through MHC-mediated antigen presentation, thereby triggering immune responses against tumors.^69^ However, while exosomes initially promote immune activation, tumors can use these exosomes to switch from immune activation to immune suppression, thereby avoiding immune clearance and contributing to therapeutic resistance. Exosomes can carry immunostimulatory molecules that amplify immune responses by promoting the activation and functionality of key immune cells, such as T cells, NK cells, and macrophages (Fig. 1).

#### 2.3.1 Activation of T cells

Exosomes derived from activated immune cells can transport cytokines, chemokines, and costimulatory signals, all of which promote the proliferation and activation of T cells, NK cells, and other key components of the immune system.^106^^,^^107^ Exosomes containing pro-inflammatory cytokines like IL-12 or IL-15 have been shown to enhance T-cell responses and recruit NK cells to sites of tumor growth.^108^, ^109^, ^110^ IL-12 plays a crucial role in enhancing T-cell responses, while IL-15 supports the survival and expansion of CD8^+^ T cells. Furthermore, exosomes from activated T cells carry TNF-α and granzyme B, contributing to the elimination of tumor cells through cytotoxic mechanisms.^111^ This action enables the immune system to better recognize and mount an attack against tumor cells, amplifying the body’s natural defense mechanisms.

#### 2.3.2 Enhancement of DC function

Exosomes can also influence DCs, enhancing their ability to present tumor antigens and stimulate T cells. TDEs often carry TGF-β and IL-10, signaling molecules that suppress DC differentiation and function.^112^ However, exosomes from activated DCs carry pro-inflammatory molecules that enhance their antigen-presenting capabilities and stimulate T cell-mediated anti-tumor responses.^112^

#### 2.3.3 Modulation of macrophage phenotype

Exosomes enriched with specific miRNAs or proteins can modulate the activity of macrophages, potentially switching them from an M2 (pro-tumorigenic) phenotype to an M1 (anti-tumor) phenotype.^113^^,^^114^ This transition not only enhances macrophage-mediated tumor cell killing but also promotes the secretion of pro-inflammatory cytokines and chemokines that recruit additional immune cells to the tumor site. This reprogramming of macrophages is a critical mechanism by which exosomes contribute to tumor immunity, as macrophages can enhance antigen presentation, secrete cytokines that activate T cells, and recruit other immune cells to the TME.

Thus, depending on the nature if the signals they carry, exosomes can either promote immune tolerance, which aids tumor survival, or stimulate immune activation, contributing to anti-tumor immunity.

### 2.4 Role of exosomes in the TME

Exosomes are integral to the dynamic interactions within the TME and contribute to various processes that facilitate tumor growth, metastasis, and immune evasion (Fig. 1). Tumor heterogeneity, a hallmark of cancer, is driven by a combination of genetic instability, differential environmental stimuli, and the stochastic interactions within the TME.^39^^,^^58^^,^^62^^,^^63^ Exosomes derived from cancer cells (CCEs) facilitate intercellular communication by transferring genetic material and proteins to neighboring cells, including healthy epithelial cells, thereby altering their phenotypic properties. This phenomenon has been observed in several cancers, such as colorectal, lung, and prostate cancers, where CCEs induce changes in the behavior of normal cells, contributing to intratumoral heterogeneity.^115^, ^116^, ^117^, ^118^

Cancer-associated fibroblasts (CAFs), key component of the TME, are also influenced by exosomes. These cells contribute to extracellular matrix (ECM) remodeling, angiogenesis, and metastasis, can be activated by TDEs carrying growth factors such as TGF-β and FGF-2.^119^^,^^120^ Studies have shown that CCEs can convert resident fibroblasts into myofibroblasts, thus promoting a fibrotic environment conducive to tumor growth and metastasis. For instance, exosomes containing TGF-β from ovarian cancer cells have been shown to induce the myofibroblast differentiation in adipose-derived mesenchymal stem cells (ADSC).^121^ Additionally, CAF-derived exosomes contribute to the metastatic process by promoting cell migration and invasion. This is achieved through the delivery of bioactive molecules like matrix metalloproteinases (MMPs) and hepatocyte growth factor (HGF), which alter the ECM and facilitate cancer cell movement.^122^

Exosomes are also integral to angiogenesis, a key feature in tumor progression. Hypoxic conditions within the TME stimulate cancer cells to release exosomes containing angiogenic factors, including miRNAs (miR-9, miR-210) and growth factors (VEGF, IL-8).^123^, ^124^, ^125^ These exosomes promote endothelial cell proliferation, migration, and the formation of new blood vessels. Exosomes derived from melanoma and breast cancer cells have been shown to active signaling pathways, such as the JAK-STAT pathway, contributing to vascular remodeling in the TME.^126^^,^^127^

Finally, exosomes play a pivotal role in metastasis by facilitating the early steps of tumor spread. They aid in epithelial-to-mesenchymal transition (EMT), a process that enables cancer cells to acquire motility and invade surrounding tissues.^128^ Exosomes derived from hypoxic cancer cells, such as those in prostate and BC, have been found to contain proteins involved in cytoskeletal rearrangement and cell-cell signaling, which enhance invasiveness and migration.^129^, ^130^, ^131^ Additionally, TDEs can prepare distant organs for metastatic colonization by influencing the premetastatic niche. For example, melanoma-derived exosomes have been shown to target lymph nodes, enhancing vascular permeability and supporting the recruitment of circulating tumor cells, which facilitates their establishment in secondary sites.^111^^,^^132^

### 2.5 Exosome-based therapies to counteract immunosuppression in cancer

Exosome-based therapies represent a promising strategy to counteract the immunosuppressive functions within the TME and improve cancer treatment outcomes. Exosomes derived from immune cells, such as NK cells, DCs, and macrophages, can be engineered to deliver immunostimulatory molecules, such as cytokines and co-stimulatory molecules, to promote the activation and expansion of effector T cells. For example, NK cell-derived exosomes contain cytolytic proteins like FasL and perforin that induce apoptosis in tumor cells, overcoming the homing difficulties faced by whole NK cells.^111^ Similarly, exosomes loaded with IL-12 or anti-PD-L1 antibodies can stimulate immune responses by reactivating exhausted T cells, blocking immune checkpoints, and overcoming the immune suppression typically exerted by TDEs, which express immunosuppressive molecules like PD-L1.^133^^,^^134^

Additionally, exosomes can be utilized as vehicles to deliver miRNAs that counteract immune suppression in the TME. MiR-155, for example, can restore immune cell activation,^101^ while the inhibition of miR-21 or miR-34a can prevent T cell exhaustion and improve the efficacy of immunotherapies.^100^, ^101^, ^102^ Exosomes can also carry Toll-like receptor (TLR) agonists, such as TLR-3 ligands, to enhance the immune response by activating DCs and macrophages, which further boosts anti-tumor immunity.^135^ This ability to modulate multiple aspects of immune function makes exosomes a versatile tool for enhancing cancer immunotherapy.

Furthermore, engineered exosomes hold potential for more targeted therapeutic applications. These exosomes can be designed to specifically target immunosuppressive cells, such as Tregs and MDSCs, by delivering antibodies like anti-CD25.^136^ By reducing the number of these immunosuppressive cells, exosomes can help improve the overall immune response against the tumor. Additionally, exosomes can be used to deliver therapeutic agents, including chemotherapeutic drugs or RNA molecules, directly to the tumor site, reducing systemic side effects and overcoming drug resistance.^137^ This dual functionality—modulating immune responses and serving as targeted drug delivery systems—positions exosome-based therapies as a powerful and adaptable platform for advancing cancer immunotherapy.

## 3 Exosome-based immunotherapy of cancer

Exosome-based immunotherapy represents a novel and promising approach to cancer treatment, using the natural immune-modulatory properties of exosomes to enhance anti-tumor immunity. By utilizing exosomes from immune cells or tumors themselves, this therapeutic strategy aims to overcome immune suppression in the TME and boost immune responses. Dendritic cell-derived exosomes (DEX) have shown great potential in priming the immune system by presenting tumor antigens, which active both cytotoxic CD8^+^ and helper CD4^+^ T cells, driving a strong anti-tumor immune response. A major advantage of DEXs is their ability to present antigens through MHC class I and II molecules, crucial for stimulating both T cell subsets.^111^ DEXs have been shown to be particularly potent in priming tumor-specific cytotoxic T lymphocyte (CTL) responses in animal models, offering a potential solution for targeting and eliminating established tumors.^138^

Moreover, DEXs help overcome resistance mechanisms within the TME, such as immune evasion by tumor cells, by enhancing antigen presentation and promoting T cell activation. This ability of DEXs to directly engage T cells and reprogram APCs can overcome immune suppression and boost the efficacy of existing therapies. DEXs induce immune responses through both direct and indirect mechanisms of antigen presentation. In the direct pathway, DEXs interact with T cells and present peptide-MHC complexes, leading to T cell activation. Indirectly, DEXs transfer these antigen-presenting complexes to APCs such as DCs, which can further prime immune responses.^139^^,^^140^ Studies have demonstrated that DEXs derived from tumor-pulsed DCs can induce robust antigen-specific CD8^+^ T cell activation and the generation of memory T cells capable of mounting long-term immune responses against tumors. This process, known as “cross-dressing,” involves the transfer of peptide-MHC complexes from DEXs to the APCs, enhancing their antigen-presenting capabilities.^139^ The therapeutic effects of DEXs have been evaluated in various cancers, including melanoma, lung cancer, and hepatocellular carcinoma (HCC), both in vitro and *in vivo*^141^, ^142^, ^143^, ^144^, ^145^, ^146^, ^147^ (Table 1).

**Table 1:** Summary of exosome-based strategies in cancer immunotherapy.

Type	Composition/method	Key findings/results	References
EXO—OVA-mAb	Exosomes from OVA-pulsed dendritic cells modified with anti-CTLA-4 antibody via lipid anchoring	Significantly inhibited tumor growth in E.G7-OVA thymoma-bearing mice by enhancing tumor-specific T-cell responses and increasing the CTLs/Treg ratio	^141^
DC-derived exosomes	DC-derived exosomes conjugated with MUC1 glycopeptide via biorthogonal crosslinking	Significantly inhibited tumor growth and prolonged survival in B16-MUC1 melanoma-bearing mice. Stimulated robust MUC1-specific IgG production, enhanced antigen presentation (MHC I/II), and promoted cytotoxic T-cell responses	^142^
DC-derived exosomes (Neoantigens)	DC-derived exosomes loaded with patient-specific neoantigens via electroporation	Demonstrated potent efficacy in B16F10 melanoma and MC-38 colorectal cancer models. Inhibited tumor growth, eliminated lung metastasis, delayed tumor occurrence, and prolonged survival through broad-spectrum T-cell and B-cell responses	^143^
DC-derived exosomes (AFP)	Exosomes from human peripheral blood-derived DCs loaded with rAAV/AFP, presenting the AFP tumor antigen	Stimulated naïve T cells to differentiate into AFP-specific CTLs and elicited significant antitumor responses against HCC	^144^
DC-derived exosomes (P47-P & AFP)	Dendritic cell-derived exosomes engineered with HCC-targeting peptide (P47-P), AFP, and immunoadjuvant N1ND-N	Enhanced antigen cross-presentation and stimulated T-cell responses in orthotopic HCC models, effectively recruiting and activating DCs	^145^
DC-derived exosomes	DC-derived exosomes enriched with AFP from lentivirus-transduced DCs expressing murine AFP	Induced strong antigen-specific immune responses, leading to tumor suppression in various HCC models through CD8^+^ T cells, with increased IFN-γ and reduced Tregs	^146^
A549 tumor lysate-pulsed DC-derived exosomes	Exosomes from cryo-activated DCs pulsed with A549 lung tumor lysate and cultured with GM-CSF, IL-4, and TNF-α	Induced significant allogeneic T-cell proliferation and cytotoxicity against A549 lung cancer cells	^147^
ESAT-6 exosomes	Exosomes from B16 melanoma cells transfected with mycobacterium tuberculosis *ESAT-6* gene, expressing TAAs and pathogenic antigen	Induced immune responses against both ESAT-6 and tumor cells, significantly suppressing tumor growth in B16 melanoma-bearing mice	^148^
CIITA-Exo	Exosomes from B16F1 melanoma cells transduced with *CIITA* gene, containing high MHC class II levels and tumor antigen TRP2	Enhanced Th1 immune responses, including TRP2-specific CD8^+^ T cells and IFN-γ secretion, significantly inhibiting tumor growth in a dose-dependent manner	^149^
CpG-SAV-exo	Exosomes from genetically engineered B16BL6 melanoma cells expressing streptavidin-lactadherin fusion protein, modified with biotinylated CpG	Enabled efficient co-delivery of tumor antigens and CpG DNA to DCs, enhancing antigen presentation and yielding stronger antitumor effects than separate exosome and CpG DNA administration	^150^
Lipo@HEV	Hybrid lipid nanovesicles combining tumor-derived exosomes with Akk-OMV and loaded with PD-L1 trap plasmid	Enhanced antitumor immunity in melanoma, breast, and colon cancers through synergistic mechanisms: DC maturation, CTL activation, and PD-L1 blockade in the tumor microenvironment	^151^
eNVs-FAP immunogenically	Exosome-like nanovesicles engineered from tumor cells expressing mutant FAP	Induced strong CTL responses against FAP^+^ CAFs and tumor cells, reprogramming the TME to inhibit tumor growth across multiple cancer models (colon, melanoma, lung, breast) by recruiting effector T cells and promoting tumor ferroptosis	^152^
EXOHSP	Exosomes from J558 tumor cells engineered to express membrane-bound HSP70, following heat shock treatment (42 °C)	Enhanced DC maturation, Th1 polarization, CD8^+^ CTL, and NK responses, resulting in improved antitumor immunity	^153^
IFN-γ-modified exosomal vaccine	Exosomes derived from RM-1 prostate cancer cells modified with an IFN-γ fusion protein anchored to the exosome surface	Significantly inhibited tumor growth, prolonged survival, and enhanced M1 macrophage differentiation, tumor-specific antibody production, and reduced metastasis. Downregulated immune suppressive factors (PD-L1, IDO1) in the TME	^154^
MVA-BN-PRO and exosome-targeted antigens	MVA immunotherapy encoding prostate-specific antigens PSA and PAP, fused to lactadherin's C1C2 domain for exosome targeting	Enhanced immune responses and antitumor efficacy; MVA-BN-PAP-C1C2 increased PAP-specific T cells and anti-PAP antibodies, improving tumor rejection. MVA-BN-PSA-C1C2 improved PSA antigenicity	^155^
spMEXO	Exosomes derived from immunogenically dying tumor cells (mitoxantrone-induced) modified with MART-1 peptide and loaded with CCL22 siRNA to inhibit the CCR4/CCL22 axis	Delayed tumor growth in PDAC models by reprogramming the TME (enhancing effector T cells, inhibiting Tregs). Combined with chemotherapy (gemcitabine, albumin-paclitaxel), it further improved therapeutic outcomes	^156^
Exo/SEA-TM	Tumor-derived exosomes from E.G7-OVA lymphoma cells, surface-modified with SEA-TM	Significantly inhibited tumor growth and prolonged survival in murine models, with CD8^+^ T cells, CD4^+^ T cells, and NK cells as the main effectors	^157^
HS-Exo	Exosomes from heat-shocked A20 B lymphoma cells (42 °C for 1 h), with increased levels of HSP60, HSP90, and immune-stimulatory molecules	Enhanced immunogenicity, inducing DC maturation, T-cell activation, and stronger antitumor immune responses in lymphoma mouse models	^158^
LEXPD-L1si	Exosomes from leukemia cells with lentivirus-mediated PD-L1 silencing (shRNA)	Induced potent anti-leukemia CTL responses, significantly inhibiting tumor growth and prolonging survival. Increased DC maturation, T-cell activation, and Th1 cytokine production (e.g., IFN-γ)	^159^
HS-TEX	Exosomes from tumor cells subjected to heat stress (42 °C for 1 hour), containing chemokines like CCL2, CCL3, and CCL4	Potently chemoattracted and activated DCs and T cells, leading to a stronger antitumor immune response and inhibition of tumor growth *in vivo*	^160^
IRF-1-Exo	Exosomes from tumor cells treated with adenovirus-expressed IRF-1 or IFN-γ, enriched with IL-15Rα and MHC-I for antigen presentation	Enhanced immunogenicity, activating tumor-specific CD8^+^ T cells and increasing IFN-γ and granzyme B production. Combined with CpG, significantly inhibited tumor growth	^161^
TAE-DC vaccine	Exosomes derived from the supernatant of tumor cells (A549, LLC) isolated by ultracentrifugation	Enhanced DC maturation, MHC cross-presentation, and tumor-specific CTL responses. Reduced PD-L1 expression on DCs and decreased Tregs in vitro. In vivo, TAE-DC vaccination inhibited tumor growth and prolonged survival by increasing CD8^+^ T cell activity and reducing Tregs in TDLNs and spleen.	^162^
EXO/IL-12	Exosomes derived from GPI-IL-12-anchored renal cancer cells (RC-2) expressing RCC-associated antigen G250 and GPI-IL-12	Enhanced T-cell proliferation and IFN-α release, inducing antigen-specific CTLs and potent in vitro cytotoxic effects	^163^
HELA-Exos	Exosomes engineered from MDA-MB-231 breast cancer cells loaded with human neutrophil elastase (ELANE) and Hiltonol (TLR3 agonist)	Induced immunogenic cell death in breast cancer cells, activated cDC1s, and triggered strong tumor-reactive CD8^+^ T cell responses. Led to potent tumor suppression in TNBC mouse models and patient-derived tumor organoids	^164^
NK 92 exosome	Exosomes derived from NK-92MI human NK cell line	Potent antitumor effects against melanoma (B16F10) cells in vitro and in vivo. Induced apoptosis in melanoma cells and inhibited tumor growth in xenograft models	^165^
CD8^+^ T Cell-derived EVs	EVs from activated CD8^+^ T cells (stimulated with tumor-specific peptides or anti-CD3/CD28 antibodies)	Interfered with fibroblastic tumor stroma, inhibiting tumor invasion and metastasis. Depleted mesenchymal tumor stromal cells (e.g., MSCs, CAFs), promoting tumor progression protection through CD8^+^ T cell infiltration and EV-mediated stromal cell depletion	^166^
M1 macrophage-derived exosomes	Exosomes from M1-polarized macrophages, rich in cytokines, proteins, and lipids mimicking parental cells	Enhanced efficacy of LCP-encapsulated Trp2 vaccine. Stimulated stronger antigen-specific CTL responses than CpG oligonucleotide adjuvants. Directed immune cells to lymph nodes, fostering a Th1-type immune response	^167^
aMT-exos	Exosomes from chimeric cells formed by fusing tumor cell nuclei (e.g., MDA-MB-231) with M1-like macrophages	Targeted primary tumors and metastases, promoting immune-stimulatory responses and tumor regression. Combined with anti-PD1 therapy, aMT-exos extended survival in metastatic and postsurgical recurrence models	^168^
M1NVs	Nanovesicles from M1 macrophages induced by LPS, prepared via extrusion and ultracentrifugation	Repolarized M2 TAMs into M1 macrophages, enhancing antitumor immune responses. When combined with anti-PD-L1 antibody, significantly suppressed tumor growth, providing a synergistic effect with checkpoint inhibition. A promising strategy for improving cancer therapy outcomes	^169^

Abbreviations: AFP, alpha-fetoprotein; Akk-OMV, Akkermansia muciniphila-derived outer membrane vesicles; aMT-exos, macrophage-tumor hybrid cell-derived exosomes; CAF, cancer-associated fibroblasts; CTLs, cytotoxic T lymphocytes; EV, extracellular vesicles; FAP, fibroblast activation protein-α; HCC, hepatocellular carcinoma; HS-TEX, heat-stressed tumor-derived exosomes; IRF-1-Exo, IRF-1-primed tumor-derived exosomes; LCP, lipid calcium phosphate; LEXPD-L1si, PD-L1-silenced exosomes; LPS, lipopolysaccharide; M1NVs, M1 macrophage-derived exosome-mimetic nanovesicles; MHC, major histocompatibility complex; MSCs, mesenchymal stem cells; MVA, modified vaccinia Ankara; NK, natural killer cell; OVA, ovalbumin; PDAC, Pancreatic ductal adenocarcinoma; RCC, renal cell carcinoma; SEA-TM, superantigen staphylococcal enterotoxin A fused with transmembrane domain; TAAs, tumor associated antigens; TAM, tumor associated macrophage; TDLNs, tumor-draining lymph nodes; Th, helper T cell; TME, tumor microenvironment; TNBC, triple-negative breast cancer; Treg, regulatory T cell.

However, a critical challenge in cancer immunotherapy is the immune suppression within the TME, where tumor cells often deploy mechanisms to evade immune surveillance. TDEs frequently carry immunosuppressive factors such as PD-L1 and TGF-β, which inhibit T cell activation and promote the accumulation of Tregs, contributing to immune tolerance and tumor escape.^148^, ^149^, ^150^, ^151^, ^152^, ^153^, ^154^, ^155^, ^156^, ^157^, ^158^, ^159^, ^160^, ^161^, ^162^, ^163^, ^164^ In some cases, TDEs may complicate immunotherapy efficacy by perpetuating immune suppression within the TME, which can limit the effectiveness of treatments. For example, the presence of PD-L1 and TGF-β in TDEs can blunt the activity of ICIs and reduce the overall response to immunotherapy. To address this, exosome-based therapies offer promising solutions by engineering exosomes to enhance their immunostimulatory properties and counteract TME-induced immune suppression.^148^, ^149^, ^150^, ^151^, ^152^, ^153^, ^154^, ^155^, ^156^, ^157^, ^158^, ^159^, ^160^, ^161^, ^162^, ^163^, ^164^ Preclinical studies have explored the use of TDEs in treating cancers such as colon cancer, breast cancer, melanoma, lung cancer, prostate cancer, pancreatic cancer, acute lymphoblastic leukemia (ALL), renal cell carcinoma, lymphoma, and HCC^148^, ^149^, ^150^, ^151^, ^152^, ^153^, ^154^, ^155^, ^156^, ^157^, ^158^, ^159^, ^160^, ^161^, ^162^, ^163^, ^164^ (Table 1). By modifying the cargo of TDEs, it may be possible to neutralize these immunosuppressive factors, ultimately enhancing immune activation and improving the overall therapeutic outcome.

Exosome derived from other immune cells, such as NK cells, T cells, and macrophages, also represent a promising strategy for enhancing cancer immunotherapy. Exosomes derived from NK cells are enriched in cytotoxic proteins such as perforin and FasL, as well as proinflammatory cytokines like tumor necrosis factor-alpha (TNF-α). These exosomes can induce apoptosis in cancer cells and inhibit tumor growth, particularly in aggressive cancers like melanoma, while sparing normal tissues from significant side effects^165^ (Table 1). Furthermore, NK cell-derived exosomes may help overcome resistance by enhancing cytotoxic activity against immune-evasive tumors, improving the effectiveness of existing immunotherapies. Similarly, exosomes derived from CD8^+^ T cells carry key cytotoxic molecules, including granzyme B and miR-298–5p, which target and deplete mesenchymal tumor stromal cells, such as CAFs and mesenchymal stem cells (MSCs), thereby reducing tumor invasiveness and metastasis. Beyond their direct cytotoxic effects, T cell-derived exosomes can enhance tumor-specific immune responses and modulate the TME, boosting the overall antitumor activity and promoting a more robust immune response against the tumor^166^ (Table 1). In addition to directly targeting tumor cells, T cell-derived exosomes may reduce immune evasion within the TME by promoting antigen recognition and boosting T cell activity, which can enhance the response to checkpoint inhibitors and other immune-based therapies.

Exosome-derived macrophages, particularly those from M1-polarized macrophages, further expand the potential of exosome-based cancer immunotherapy^167^, ^168^, ^169^ (Table 1). M1 macrophage-derived exosomes are packed with bioactive molecules, including proinflammatory cytokines and miRNAs that stimulate antitumor immune responses by enhancing T cell activation and promoting a more inflammatory microenvironment. These exosomes can also reprogram tumor-associated macrophages (TAMs), repolarizing immunosuppressive M2 macrophages into a more cytotoxic M1 phenotype. Additionally, M1 exosomes exhibit tumor-homing properties, improving the efficacy of cancer vaccines and immune checkpoint therapies, while also reducing tumor growth and metastasis.^167^, ^168^, ^169^ Thus, M1-derived exosomes could be particularly valuable for promoting immune activation and combating immunosuppressive environments that often limit the efficacy of cancer immunotherapy. Given their ability to selectively target and influence key immune cells within the TME, M1-derived exosomes represent a versatile and powerful strategy to enhance the efficacy of cancer immunotherapies.

While exosome-based immunotherapy has been explored in various cancers, its application in bladder cancer remains limited. A recent study demonstrated that EVs from murine MB49 bladder cancer cells can induce CD8^+^ *T*-cell-mediated tumor suppression and enhance immune infiltration, highlighting their potential as an immunotherapeutic strategy.^170^ However, the immunosuppressive nature of the TME in bladder cancer may also limit the effectiveness of exosome-based therapies, emphasizing the need for careful optimization to overcome potential resistance. Further research is needed to evaluate their clinical applicability.

## 4 Immunotherapy for BC

BC is a highly immunogenic malignancy, and immunotherapy has become a key strategy in its treatment, especially for non-muscle invasive disease (NMIBC). Bacillus Calmette–Guérin (BCG) therapy, a live attenuated strain of *Mycobacterium bovis*, has been one of the first and most widely adopted immunotherapies for BC since 1976.^171^ Administered directly into the bladder, BCG induces a robust local immune response through the activation of both innate and adaptive immune systems.^172^ Upon instillation, BCG interacts with bladder mucosa, stimulating the recruitment of immune cells such as macrophages, DCs, and T lymphocytes.^172^, ^173^, ^174^ This triggers the release of pro-inflammatory cytokines, including interferon-gamma (IFN-γ), TNF-α, and various interleukins, which enhance immune cell activation.^175^^,^^176^ These cytokines promote the activation of cytotoxic T cells, which are critical for targeting and eliminating cancer cells.^175^ Additionally, BCG may stimulate the upregulation of immune checkpoints, thereby increasing the immune system’s ability to recognize and attack tumor cells.^177^ The combined effect of these immune responses contributes to the destruction of both primary tumors and potential metastases, making BCG a cornerstone in the treatment of NMIBC.

The introduction of ICIs in 2016 has transformed the treatment landscape for advanced, metastatic, and cisplatin-resistant muscle-invasive bladder cancer (MIBC).^178^ However, despite the promising results of ICIs targeting immune checkpoint molecules, the overall response rate remains limited, highlighting the challenges in optimizing immunotherapy for BC.^179^ PD-1 and PD-L1 are critical immune checkpoint molecules that play a central role in immune evasion by tumors, including BC. Under normal physiological conditions, PD-1 is essential for maintaining immune homeostasis by preventing excessive immune activation and autoimmunity.^180^ In the TME, the interaction between PD-1 on activated T cells and PD-L1 on tumor cells results in immune suppression, allowing cancer cells to evade immune surveillance and promote tumor progression.^181^ ICIs targeting PD-1 (e.g., pembrolizumab, nivolumab) and PD-L1 (e.g., atezolizumab, avelumab, durvalumab) have revolutionized the treatment of metastatic BC, particularly in patients with cisplatin-resistant disease.^182^ These therapies have shown durable responses and have been approved by the U.S. Food and Drug Administration (FDA) for use in second-line settings after failure of platinum-based chemotherapy.^181^ However, despite these advances, the objective response rate remains around 20%, indicating that only a subset of patients benefits from immune checkpoint blockade (ICB).^182^ Several factors contribute to resistance to ICB therapies, including the immunosuppressive TME and the heterogeneity of PD-L1 expression.^183^ Although PD-L1 expression is generally considered a biomarker for predicting ICI efficacy, its expression on tumor cells is not always consistent, leading to a variable response to therapy.^184^ Additionally, irAEs are a concern with ICB therapy, with clinical responses sometimes correlated with the development of irAEs.^184^ While these events are generally less frequent with PD-1/PD-L1 inhibitors, their occurrence can complicate treatment regimens and affect the quality of life for patients.

CTLA-4 is another immune checkpoint receptor that plays a crucial role in regulating immune responses.^185^ While essential for T cell activation, CTLA-4 functions primarily as a negative regulator, dampening the activity of T cells to prevent excessive immune reactions.^186^ The mechanism of CTLA-4′s inhibitory effect is not fully understood, but it is known to suppress immune responses by inhibiting intracellular signaling through its cytoplasmic tail. CTLA-4 has been proposed as an alternative target for cancer immunotherapy, with the hypothesis that blocking CTLA-4 could enhance the immune system's ability to recognize and attack tumor cells. Research into anti-CTLA-4 antibodies has shown promising results in improving immune responses in cancer treatments, including BC, by overcoming immune suppression and promoting stronger anti-tumor immunity.^187^

## 5 Exosome in BC

### 5.1 Exosomes as non-invasive diagnostic tools in BC

Exosomes are emerging as valuable non-invasive biomarkers for BC, providing a promising alternative to traditional biopsy technique. The cargo within these vesicles reflects the pathological status of the cells from which they originate, providing valuable insights into tumor characteristics.^188^ Urinary exosomes, in particular, are especially useful for diagnosing BC, as they allow for the analysis of tumor-specific molecular signatures without invasive procedures.

Urinary exosomes are a rich source of miRNAs that are differentially expressed in BC patients compared to healthy individuals. Several studies have demonstrated significant alterations in the miRNA profiles of exosomes, making them useful for diagnosis and staging. For instance, miR-146b-5p, miR-155–5p, miR-138–5p, and miR-200a-3p are frequently deregulated in BC tissues and exosomes, with distinct expression patterns that vary depending on cancer stage.^189^, ^190^, ^191^ A study by Cheng et al. showed that miRNAs such as miR-200a are particularly elevated in MIBC compared to NMIBC, highlighting their potential for distinguishing between these two subtypes.^191^ Additionally, specific miRNAs in exosomes have been shown to detect early-stage BC, including non-muscle-invasive cases that are difficult to identify with traditional methods like urine cytology. For example, miR-93–5p and miR-375 are upregulated in urine exosomes from BC patients and are associated with poor prognosis high-grade tumors.^192^^,^^193^ Studies have also demonstrated that miR-194–5p and miR-155 are highly expressed in urinary exosomes of patients with recurrent BC, making them potential markers for recurrence monitoring.^194^, ^195^, ^196^ Furthermore, miR-21–5p has been found to be upregulated in the urinary exosomes of BC patients, with an area under the curve (AUC) of 0.900, sensitivity of 75%, and specificity of 95.8% for detecting uroepithelial cancer.^196^ These findings suggest that miRNAs in exosomes can be used not only for early detection but also for predicting tumor aggressiveness and recurrence.

Exosomal proteins also play a crucial role in the molecular characterization of BC. Proteomic studies of BC-derived exosomes have revealed differentially expressed proteins that provide diagnostic and prognostic information. For example, Welton et al. identified 353 proteins from exosomes isolated from BC cell lines and patient urine samples, including MUC1, CD44, integrins, and CD7, which were significantly overexpressed in BC exosomes.^197^ These proteins are involved in important processes such as EMT, cell migration, and tumor progression. MUC1, a cell surface glycoprotein, is often aberrantly expressed in BC and is linked to tumor cell survival and resistance to apoptosis.^198^ CD44, a cell adhesion molecule, is known to play a role in cancer cell adhesion, migration, and invasion, with its overexpression associated with more aggressive cancer phenotypes.^199^ Integrins, particularly integrin β1 and integrin α6, mediate cancer cell migration and invasion and are found at elevated levels in BC-derived exosomes.^200^ TACSTD2, involved in cell adhesion, is significantly higher in BC exosomes compared to healthy controls, indicating its potential as a diagnostic biomarker for advanced disease.^201^ Additionally, proteomic analysis of urinary exosomes has highlighted proteins such as α1 antitrypsin and H2B1K, which can distinguish urothelial cancer from non-cancerous tissues.^202^ These proteins show diagnostic accuracy superior to traditional test like the occult blood test, making them valuable for monitoring therapy response and disease progression.

Further studies have highlighted the overexpression of MAGE-B4 and NMP22 in exosome-derived samples from BC patients, emphasizing their potential as diagnostic markers.^203^ In a study by Chen et al.,^201^ the analysis of urinary exosome proteins revealed 24 proteins with notable concentration changes compared to the control group with AUC values ranging between 0.702 and 0.896. Notably, TACSTD2 levels were significantly elevated in urinary exosomes, being 6.5 times higher in individuals with BC, reinforcing its potential as a novel biomarker for early diagnosis and prognosis.

In addition to miRNAs and proteins, exosomes also carry mRNAs and long non-coding RNAs (lncRNAs), which are important components in exosomal diagnostics. For example, GALNT1 and LASS2 mRNAs have been identified as exclusively present in the urinary exosomes of BC patients.^204^ Moreover, lncRNAs like SPRY4-IT1, MALAT1, and PCAT-1 have been found to be differentially expressed in BC exosomes.^205^ These lncRNAs, particularly SPRY4-IT1, MALAT1, and PCAT-1, exhibit strong sensitivity and specificity, with their combined use offering a higher AUC (0.813) compared to traditional tests like urine cytology.^205^

Exosomal DNA, particularly cell-free DNA (cfDNA) from urinary exosomes, provides further valuable insights into genetic mutations associated with BC, facilitating non-invasive genetic screening. For instance, *FGFR3* mutations have been detected in exosomal DNA, enabling real-time tracking of tumor mutations and monitoring therapy response, especially in patients undergoing BCG treatment.^206^ Exosomal cfDNA analysis can also detect minimal residual disease (MRD) following treatment, which is essential for early recurrence detection, particularly in BC where relapse is common.^207^ Exosomal cfDNA has several advantages over traditional tumor biopsies, including its ability to capture the genetic heterogeneity of tumors without invasive procedures.^208^ Recent studies have demonstrated that cfDNA in exosomes can monitor genetic alterations that may lead to therapy resistance, including resistance to chemotherapy and immunotherapy.^208^ Ellinger et al.^209^ conducted a study using methylation-specific PCR to evaluate the methylation status of *GSTP1, TIG1*, and *APC* genes in serum samples from BC patients undergoing cystectomy. The analysis revealed hypermethylation in 80% of the samples, demonstrating a high specificity of 93%. Additionally, the presence of two short DNA fragments (124 bp *PTGS2* and 271 bp *RPRM*) in the serum DNA was assessed, and the apoptosis index (ratio of 124 bp to 271 bp fragments) was able to reliably differentiate BC from benign prostate hyperplasia, with 96% sensitivity and 62% specificity.^210^

### 5.2 The role of exosomes in immune modulation and BC pathogenesis

Recent studies have highlighted the critical role of exosomes in mediating cellular communication and driving the pathogenesis of BC. Exosomes secreted by BC cells contain a complex cargo of proteins, lipids, and genetic materials (including miRNAs and lncRNAs), which can be transferred to other cells within the TME or distant sites, thereby influencing tumor progression, drug resistance, and metastasis. Regarding immune modulation, exosomal miR-21 secreted by BC cells induces macrophage M2 polarization, promoting an immunosuppressive TME that favors tumor growth. MiR-21 achieves this by inhibiting tumor suppressors such as PTEN and PDCD4, activating the PI3K/Akt signaling pathway, and contributing to carcinogenesis.^211^ Exosomes from BC cells have also been shown to impair NK cell function. For instance, T24 cell-derived exosomes reduce NK cell viability and cytotoxicity by inhibiting the expression of key functional receptors such as NKG2D, NKp30, and CD226. High-throughput sequencing identified miR-221–5p and miR-186–5p as critical miRNAs in these exosomes, which interfere with the stability of mRNAs involved in NK cell activation.^212^

There is substantial evidence indicating the role of exosomes in the pathogenesis of BC, including cell proliferation, apoptosis, invasion, angiogenesis, and metastasis. Exosomes mediate these effects through their cargo, including miRNAs, proteins, and lipids, by affecting various signaling pathways. MiRNAs are selectively packaged into exosomes and delivered to recipient cells, where they regulate tumor biology. Exosomal miR-4644, upregulated in the plasma of BC patients, enhances tumor growth by downregulating *UBIAD1*, a gene involved in tumor suppression.^213^ Exosomal miR-663b has also been linked to EMT by targeting *ERF*, a gene involved in cell differentiation regulation and cell proliferation.^214^

Exosomes also influence cancer metastasis and drug resistance. Exosomes derived from CAFs transport miR-148b-3p into BC cells, enhancing metastatic behavior and inducing resistance to chemotherapy drugs such as paclitaxel and doxorubicin, both in vitro and *in vivo*.^215^ TDEs also play a role in tumor progression and angiogenesis. For example, miR-1247–3p, highly expressed in exosomes from BC cells, promotes angiogenesis by targeting FOXO1, a gene involved in cell survival and proliferation.^216^ Moreover, Yuan et al. demonstrated that elevated exosomal miR-17–5p contributes to BC progression by targeting ARID4B and modulating the immune system.^217^ Other miRNAs, such as miR-21, miR-133b, and miR-93–5p, also affect cell proliferation.^218^^,^^219^

In addition to miRNAs, exosomes also carry proteins that play crucial roles in the pathogenesis of BC. For instance, the protein EDIL-3/Del1, secreted by bladder tumor cells within exosomes, accelerates tumor progression by promoting cell adhesion and migration.^220^ CAFs secrete exosomes containing LINC00355, which contribute to tumor growth, invasion, and therapeutic resistance by modulating the tumor microenvironment. The Wnt/β-catenin signaling pathway, activated by exosomal miRNAs such as miR-375–3p, also impairs tumor suppression mechanisms and promotes chemotherapy resistance.^221^, ^222^, ^223^ Proteomics studies have further elucidated the role of exosomal proteins in BC progression. For example, the expression of KRT6B in BC exosomes correlates with clinical stage, tumor invasion, and metastasis by regulating macrophage differentiation.^224^ Similarly, Jiang et al. showed that exosomes derived from BC cell lines, such as MB49, promote tumor growth by activating the PTEN/AKT/STAT3 signaling pathways in the TME.^211^ GFTA1 induces HBP-related metabolic reprogramming and SerRS O-GlcNAcylation in endothelial cells, influencing angiogenesis.

LncRNAs are another class of genetic material packaged into exosomes, and due to their role in regulating cellular processes, they are essential for cancer metastasis and pathogenesis.^225^ LncRNAs such as LINC00960 and LINC02470 have been identified as key regulators of BC progression.^129^ These lncRNAs promote the aggressiveness of recipient low-grade BC cells by activating EMT signaling pathways, including β-catenin, Notch, and Smad2/3.^129^ The transfer of these lncRNAs via exosomes facilitates the transition of low-grade tumor cells into a more aggressive, metastatic phenotype, suggesting their potential as biomarkers for aggressive disease and as therapeutic targets. Additionally, the lncRNA LINC01133 has been implicated in inhibiting BC progression by modulating the Wnt signaling pathway. Exosomal LINC01133, derived from normal urothelial cells, suppresses BC cell growth and invasiveness by downregulating components of the Wnt/β-catenin pathway, which is known to drive cancer metastasis.^226^

### 5.3 Exosome based therapy of BC

Exosome-based therapy is being actively explored for the treatment of various disorders, including BC. Recent studies have highlighted the potential of exosome-derived miRNAs in modulating key pathways involved in BC progression, metastasis, and drug resistance, offering new therapeutic opportunities. Liu et al.^227^ demonstrated that exosomes derived from ADSC umbilical loaded with miR-138–5p inhibited BC cell migration, invasion, and proliferation, and suppressed tumor growth in a mouse model. This suggests their potential as an effective, non-toxic delivery system for miRNA-based therapy. Similarly, Jia et al.^228^ showed that miR-139–5p-loaded exosomes from human umbilical cord mesenchymal stem cells (hUCMSCs) downregulated PRC1, inhibited BC progression in vitro and *in vivo.* Cai et al.^229^ also found that exosomal miR-133b, which is downregulated in BC tissues, inhibited cell proliferation and promoted apoptosis by targeting DUSP1. In another study, Fu et al.^230^ demonstrated that exosomal miR-19b-1–5p, derived from bone marrow mesenchymal stem cells (BMSCs), suppressed BC cell proliferation and inhibited tumor growth by targeting the non-receptor protein tyrosine kinase ABL2. The therapeutic potential of MSC-derived exosomes has been further supported by the work of Jia and Cai et al.,^229^^,^^231^ which showed that exosomal miR-9–3p and miR-139–5p, respectively, suppressed BC cell proliferation and migration by targeting key molecules like ESM1 and PRC1 that are critical for tumor cell survival and metastasis.

Beyond miRNAs delivery, exosomes also have the capacity to modulate the TME. Shan et al. demonstrated that exosomes derived from CAFs, containing the miRNA miR-148b-3p, promoted BC progression by targeting PTEN and activating the Wnt/β-catenin pathway, which promotes BC metastasis, EMT, and chemoresistance.^215^ Targeting exosomal miR-148b-3p could be a potential strategy for reversing drug resistance and inhibiting tumor progression in BC. Additionally, Li et al.^232^ showed that exosomal miR-375–3p suppressed tumor growth by targeting the Wnt/β-catenin pathway, indicating that exosome-mediated delivery of miR-375–3p could be a strategy for inhibiting BC proliferation and metastasis.

## 6 Clinical trial of exosome based immunotherapy and future role in BC

Exosomes have gained significant attention in clinical applications due to their versatile roles in cancer diagnosis and treatment. These EVs are being explored for their potential as biomarkers for cancer diagnosis, prognosis, and treatment efficacy, as well as for their ability to deliver therapeutic agents in a targeted manner. A search of “ClinicalTrials.gov” using the terms “exosome” and “exosomes” in the context of cancer yields approximately 90 results, highlighting their growing clinical relevance. Due to their non-invasive nature and ease of collection, exosomes have become particularly valuable as biomarkers, with approximately 93% of studies focusing on their diagnostic and monitoring applications, rather than direct therapeutic uses. Unlike traditional tissue biopsies, exosomes can be isolated from readily accessible body fluids such as blood, urine, or saliva, making them an attractive tool for real-time, non-invasive cancer diagnostics. Exosomes are being studied as biomarkers in a wide range of cancers, including lung, breast, prostate, gastric, colorectal, bladder, and thyroid cancers, where they are being investigated for roles in diagnosis, monitoring disease progression, and assessing treatment efficacy. Additionally, exosomes are being explored as therapeutic products in various cancers, including rectal cancer, colon cancer, pancreatic cancer, lung cancer, and acute myeloid leukemia (AML).

Although less prevalent, the use of exosomes in cancer immunotherapy is an emerging area of research. Searching for “immunotherapy + exosome” on ClinicalTrials.gov yields five studies, with applications ranging from exosome-based immune biomarkers for non-Hodgkin B-cell lymphoma (NCT03985696), to evaluating immunotherapy efficacy in HCC, renal cell carcinoma, and gastrointestinal tumors (NCT05575622, NCT05705583, NCT05427227). One study is also exploring the use of exosomes derived from TLR4L- or IFN-γ -maturated DCs in patients with unresectable NSCLC who have shown a response to induction chemotherapy (NCT01159288).

A review of the literature reveals several clinical trials investigating exosome-based immunotherapy for treating various conditions. For instance, Morse et al. assessed the safety and efficacy of autologous DEXs loaded with MAGE tumor antigens in patients with NSCLC, demonstrating long-term disease stability and immune activation in some patients.^233^ Similarly, Escudier et al.^234^ evaluated autologous DEXs pulsed with MAGE-3 peptides in stage III/IV melanoma patients, confirming the safety of exosome administration. Narita et al.^235^ explored exosome-derived monocyte-derived dendritic cells (moDCs) pulsed with the SART1 peptide for treating esophageal squamous cell carcinoma, while Dai et al.^236^ used ascites-derived exosomes (Aex) in combination with GM-CSF for colorectal cancer (CRC) immunotherapy.

In BC, four clinical studies are investigating exosomes, primarily focusing on their potential as biomarkers. These include analyses of exosome glycosylation patterns in the urine (NCT04960956), expression of lncRNA-ELNAT1 in urine-derived exosomes (NCT05270174), and urine exosomal RNA expression (NCT06193941). Additionally, a clinical trial, titled "An Open, Dose-escalation Clinical Study of Chimeric Exosomal Tumor Vaccines for Recurrent or Metastatic Bladder Cancer" (NCT05559177), initiated in 2022, aimed to explore the therapeutic potential of exosomes. This early phase 1 trial focused on assessing the safety, tolerability, and optimal dosage of a personalized chimeric exosome vaccine derived from APC-tumor chimeric cells. However, no results have been reported to date.

Exosome-based therapies can hold significant potential when integrated with existing immunotherapies, including ICIs, vaccines, and ACT. One promising strategy is combining exosomes with ICIs, such as anti-PD-L1 or anti-CTLA-4 antibodies, to overcome immune evasion mechanisms and improve therapeutic efficacy.^133^ Exosomes can deliver immune-stimulatory molecules that block the PD-1/PD-L1 axis, which is commonly exploited by tumors to escape immune surveillance.^237^ By delivering these molecules directly to the tumor site, exosomes may enhance the effects of ICIs, leading to better activation of T cells and greater anti-tumor immune responses.^237^

In addition, exosomes can be used to enhance the effectiveness of ACT by delivering tumor-associated antigens (TAAs) or stimulating DCs to improve T cell priming and expansion.^133^ Exosome-based vaccines, particularly those loaded with tumor-specific antigens, can be used in combination with ACT to enhance the activation of tumor-specific T cells.^69^ This could provide a more robust immune response, potentially overcoming resistance mechanisms in solid tumors and improving the success rates of ACT. For example, exosomes derived from tumor cells or DCs loaded with specific antigens can be used to "train" T cells, enabling them to recognize and target tumor cells more effectively.^238^

Moreover, exosomes have the ability to carry miRNAs and proteins that can directly modulate the immune response. By loading exosomes with specific miRNAs, such as miR-155 or miR-34a, it may be possible to restore immune cell activation and prevent T cell exhaustion, which is often a challenge in immunotherapy-resistant tumors.^100^^,^^102^ The combination of exosome-based delivery systems with ICIs or ACT could also address the issue of tumor heterogeneity and facilitate targeted drug delivery, improving the effectiveness of immunotherapies in challenging solid tumor environments.

Immunotherapy has been extensively studied in BC, with nearly 140 clinical trials conducted to date. About 12% of these focus on BCG therapy, an established immunotherapy still in use today to enhance treatment efficacy. Other studies investigate the use of ICIs and their combinations with radiotherapy or other therapies. For example, one trial (NCT03389438) is exploring autologous cellular immunotherapy, where a patient’s T cells are cultured and then re-infused to stimulate an anti-tumor immune response. However, no clinical trials have yet been registered using the term "immunotherapy + exosome" specifically for BC.

Recent preclinical studies suggest that exosomes hold significant potential for the diagnosis, prognosis, and treatment of BC. However, clinical trial data remains limited due to mixed efficacy results, concerns over biosafety, and the need for further optimization before exosomes can be effectively translated into clinical practice.^236^^,^^239^^,^^240^ More research is needed to better understand the mechanisms of action of exosomes, particularly their effects on immune modulation, including T cells, NK cells, and the specific pathways leading to their activation. Furthermore, understanding how exosomes interact with the TME is crucial for their effective clinical application. Targeted therapies that optimize the use of exosomes are also a promising avenue requiring further investigation. Several factors also need to be optimized before exosome-based therapies can be used in clinical settings, such as exosome isolation, optimal dosage, timing of administration, and other procedural aspects.^240^

Personalized medicine has recently expanded, with many studies focusing on its potential to improve treatment outcomes. In this context, personalized exosome-based immunotherapies can enhance treatment efficacy. This approach involves isolating a patient's own exosomes, typically derived from tumor cells or immune cells, and modifying them to carry specific tumor antigens or immunomodulatory molecules. These modified exosomes allow the immune system to recognize and target the patient’s unique cancer cells with a tailored response, effectively creating a "personalized vaccine" that stimulates the immune system to target the specific tumor profile.

In conclusion, despite the challenges, exosome-based immunotherapy for BC remains a promising approach. Preclinical studies indicate its potential to become a standard treatment for various disorders in the future, but it is still in the early stages of development. Further preclinical research is crucial to addressing existing concerns and answering key questions, which will be essential for accelerating the translation of this promising therapy from laboratory to clinical practice (Fig. 1).

## Declaration of competing interest

The authors declare that they have no known competing financial interests or personal relationships that could have appeared to influence the work reported in this paper.
